# Impact of nutritional and multiple micronutrients supplementation to lactating mothers 6 months postpartum on the maternal and infant micronutrient status: a randomised controlled trial in Delhi, India

**DOI:** 10.1017/S1368980024001824

**Published:** 2024-09-26

**Authors:** Rukman Manapurath, Ranadip Chowdhury, Ravi Prakash Upadhyay, Beena Bose, Sarita Devi, Pratibha Dwarkanath, Anura V Kurpad, Nita Bhandari, Sunita Taneja

**Affiliations:** 1 Society for Applied Studies, New Delhi, India; 2 Centre for International Health, University of Bergen, Bergen, Norway; 3 Department of Physiology, St John’s Medical College, Bengaluru, India

**Keywords:** Lactating mothers, Micronutrient deficiency, Micronutrient concentrations, Nutritional supplementation

## Abstract

**Objectives:**

To assess the impact of nutritional and multiple-micronutrient supplementation to lactating mothers on the micronutrient status of mother–infant dyad at 6 months of age postnatally.

**Design:**

This study was a trial that aimed to investigate the impact of maternal nutritional supplementation on infant growth. A secondary objective was to assess the effect on the micronutrient status of mother–infant pairs. The intervention group mothers received snacks with 600 kcal energy, 20 g protein and daily micronutrient tablets.

**Setting:**

Blood samples were collected from both mothers and infants at 6 months.

**Participants:**

The participants in this study were mother–infant pairs. The micronutrient status of these pairs was assessed through blood samples, focusing on vitamins A, D, B_12_, ferritin, Zn and folate.

**Results:**

Micronutrient analysis of serum samples from 600 mother–infant pairs showed that mothers in the intervention group had higher levels of serum ferritin (mean difference (MD) 14·7 ng/ml), retinol (MD 0·6 μmol/l), folate (MD 3·3 ng/ml) and vitamin D (1·03 ng/ml) at 6 months postpartum. Additionally, the supplementation was associated with a higher mean ± sd of serum ferritin (MD 8·9 ng/ml) and vitamin A (MD 0·2 μmol/l) levels in infants at 6 months.

**Conclusions:**

The study found that supplementing maternal nutrition with additional dietary and micronutrient intakes during lactation improved maternal micronutrient status and slightly increased ferritin and vitamin A levels in infants at 6 months. The findings highlight the importance of nutritional interventions for improving the micronutrient health of mother–infant pairs, with significant public health implications.

Trial registered at www.clinicaltrials.gov (CTRI/2018/04/013095).

Micronutrients comprising of vitamins and minerals are essential for maintaining normal cellular and molecular functions of the body, despite being required in smaller quantities^(1)^. However, a significant proportion of the global population, spanning across all age groups, suffers from micronutrient deficiency^(2)^. This problem is particularly prevalent in low- and -middle income countries (LMIC)^(3)^, where factors such as poor quality of food, lack of dietary diversity, infections and associated inflammation contribute to the deficiency^(4)^. One of the major consequences of micronutrient deficiency is anaemia. As per WHO, non-pregnant women, lactating mothers, have the highest burden of anaemia across the globe. During lactation, meeting the increased nutritional demands required to support infant growth solely through diet may be challenging^(3)^. While nutritional supplements in the form of healthy snacks could potentially help meet these demands, current evidence on their measurable benefits is limited.

Research has shown that micronutrient deficiencies may lead to impaired physical growth and psychomotor development in children as well as adults^(5–8)^. However, studies focusing on interventions for addressing micronutrient deficiencies have generally overlooked the postpartum period, which is a critical phase for maternal and infant health^(9)^. It is important to note that micronutrient deficiencies such as Fe, vitamin A, B vitamins and Zn often co-exist, as they are predominantly caused by a lack of a balanced diet^(10)^. This suggests that supplementing these vitamins and minerals could potentially improve the micronutrient status of the mother–infant dyad. However, there is a lack of evidence that supports the beneficial effect of nutritional interventions such as dietary and micronutrient supplements on infant micronutrient concentrations during the early stages of growth, except for Fe and folic acid^(11)^.

Earlier studies have primarily focused on comparing individual micronutrients or examining pregnancy outcomes, overlooking the critical postpartum period^(12,13)^. To address this research gap, our current analysis of the IMPRINT trial aimed to assess the effect of the same interventions on the micronutrient status of the mother–infant dyads. We hypothesise that ensuring intake of additional requirements for macro and micronutrients through additional dietary and multiple micronutrient (MMN) supplements among lactating mothers for the first 6 months will improve the maternal micronutrient status, subsequently benefiting infant micronutrient status at 6 months of infant age in comparison with routine care. The published trial found that maternal supplementation in the first 6 months postpartum led to a small, non-significant improvement in infant linear growth at 6 months of age in addition to improvements in maternal BMI (mean difference 0·37 kg/m^2^ (95 % CI: 0·09, 0·64)) and Hb concentrations (mean difference 0·37 g/dl (95 % CI: 0·19, 0·56))^(14)^.

## Methods

### Study setting, design, participants and randomisation

This analysis was conducted on blood samples collected from the IMPRINT study^(14)^. The study aimed to evaluate the impact of additional dietary and 80–100 % recommended dietary allowances MMN supplementation among lactating mothers for the first 6 months postpartum on infant linear growth. The study was carried out from 9 May 2018 to 31 May 2019, conducted in low-resource settings of urban Delhi, India. The study used a web-based randomisation list with variable blocks to assign mother–infant dyads to intervention or control groups, collected baseline socio-economic and anthropometric data and minimised contact between the delivery, counselling and outcome teams. Detailed information about the study design and the main findings have been published previously^(14)^. Briefly, the mother–infant dyad was screened within 7 d of birth. As per the recommended dietary allowances by ICMR – NIN, 2020, mothers in the intervention group received snacks to be consumed daily. These snacks provided 600 kcal of energy and 20 g protein. Of the total energy, 25–30 % (150–180 kcal) was derived from fats and 13 % (80 kcal) from protein. This nutritional supplementation was designed to meet the additional nutrient requirements of the mothers in the intervention group^(15)^. The food supplements were prepared by a for-profit organisation, Hungry Foal, and provided in the form of locally acceptable snacks. These snacks were pretested for acceptability among women in the study population before study initiation. Mothers were given the snack of their choice, with an option to change their preference at the time of weekly replenishment. In addition to the snack, micronutrient supplementation was provided for a period of 180 d as daily tablets that provided 80–100 % of the recommended dietary allowances of various vitamins and minerals. The composition of the MMN tablets donated by The Vitamin Angel Alliance, Inc. (Vitamin Angels) from California was similar to the UNICEF/WHO/United Nations University international MMN preparation^(16)^. Data on compliance with the nutritional and micronutrient supplements among the intervention-group mothers has already been published previously^(14)^.

Six millilitres of venous blood from the infant and eight millilitres from the mother were collected to measure micronutrient status at 6 months. The samples were centrifuged at 3000 rpm for 15 min, and serum samples were stored at the Central Research Laboratory, Society for Applied Studies, Delhi, India, in the –80°C deep freezer before being sent for analysis. All micronutrient analyses were performed at the St. Johns Research Institute Bangalore, India.

All mothers provided written informed consent. Approval for the study was obtained from ethics committees at the Society for Applied Studies, India. The trial was registered at www.ctri.nic.in (CTRI/2018/04/013095).

### Outcome

The concentrations of ferritin, vitamin B_12_, folate and vitamin D were measured by the chemiluminescence principle using the Cobas E411 immunoassay analyser (Roche Diagnostics, USA). Lyphochek Immunoassay trilevel controls (Biorad, USA) were used as quality controls for ferritin, vitamin B_12_ and folate, while Roche-specific quality controls were used for vitamin D measurements. The intra- and inter-assay CV were below 1·0 % and 5·0 % for all the controls, respectively.

Serum Fe and Zn concentrations were determined by flame atomic absorption spectrometry (Atomic absorption spectroscopy iCE 3000 series, Thermo Fisher Scientific). Commercially available Tritrisol standards (Merck) were used for generating the standard curve with the flame-type air-acetylene, oxidising both elements. The recovery of the reference material was between 95 and 105 % with an intra- and inter-assay CV of 1·0 % and 4 %, respectively.

The vitamin A analysis was performed using LC with MS. Serum samples were spiked with stable isotopically labelled (d4 retinyl acetate, CIL) internal standards and deproteinised with a mixture of methanol containing 1 % acetic acid in water. Each sample was vortexed thoroughly and centrifuged. Chromatographic separation was achieved using Poroshell 120, EC-C18 column (Agilent Technologies), and MS analysis was performed in a dynamic multiple reaction monitoring based method in positive electrospray ionisation mode. Concentrations of each vitamin were calculated using analyte-specific calibration curves generated with their respective stable isotopically labelled internal standards.

#### Definitions

Maternal micronutrient proportion deficient calculated with cut-off defined as ferritin <15 ng/ml^(17)^, vitamin A < 0·7 μmol/l^(18)^, vitamin B_12_ < 203 pg/ml^(19)^, vitamin D < 12 ng/ml, folate < 4 ng/ml^(20)^ and Zn < 66 µg/dl^(21)^. Infant micronutrient proportion deficient was defined with cut-off as ferritin < 12 ng/ml^(22)^, vitamin A < 0·7 μmol/l^(23)^, vitamin B_12_ < 203 pg/ml^(19,23)^, vitamin D < 12 ng/ml, folate < 4ng/ml^(23)^ and Zn < 65 µg/dl^(21)^.

### Statistical analysis

With a total sample size of 600 children analysed for micronutrient status, we had at least 90 % power to detect differences between the two groups of 0·3 sd in means and 20 % in proportions deficient with 95 % CI. The analysis was carried out using Stata version 16·1. Continuous variables were presented as median with interquartile range or mean with sd, while categorical variables were reported as counts (N) and percentages (%). Any values that exceeded three interquartile ranges above the 95th percentile (or below the fifth percentile) were identified as extreme outliers and excluded from any analysis^(24)^. We used a generalised linear regression model of the Gaussian family with an identity-link function for continuous outcomes. For estimating the effect of supplementation on the categorical outcome of micronutrient concentrations (deficient *v*. sufficient), we used a generalised linear regression of the binomial family with a log-link.

We performed a purposive selection of variables at baseline to identify potential confounders for adjustment in the model, and their impact on the effect size was assessed through univariate analysis. Variables that brought at least a 15 % change in effect size were considered potential confounders for adjustment^(25)^. These variables included gestational age, length-for-age z-score (LAZ), BMI *z*-score, wealth quintiles and maternal BMI at baseline for infant micronutrient concentrations and wealth quintiles and maternal BMI at baseline for maternal micronutrient concentrations. All applicable statistical tests were two-sided and performed using a 5 % significance level.

## Results

**Fig. 1:**
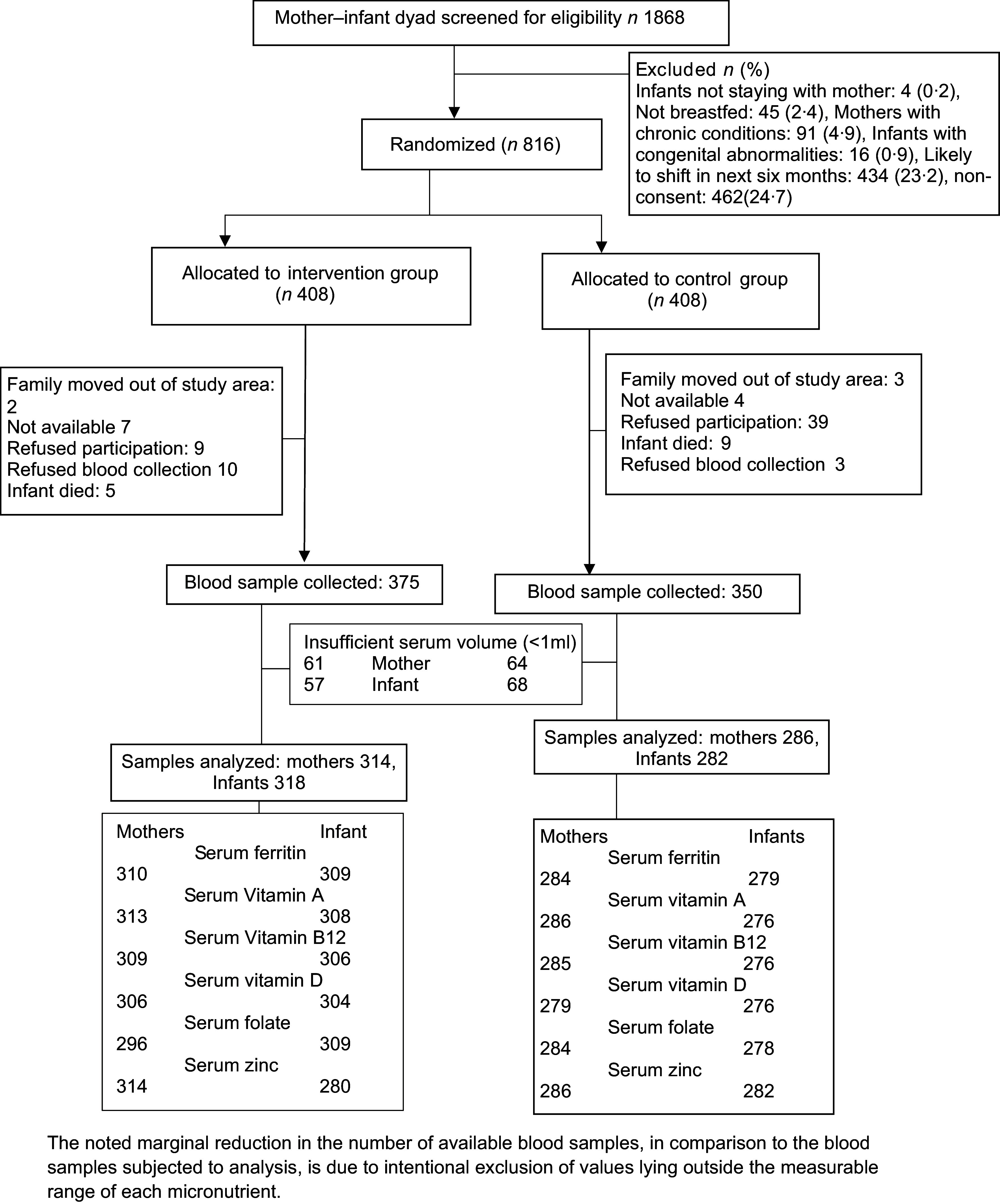
Trial profile

Out of 1868 mother–infant pairs screened for eligibility, 816 pairs were assigned to either the intervention or control group, resulting in 408 pairs in each cohort. Blood samples were collected from 375 and 350 mother–infant dyads from the intervention and control group, respectively (Fig. 1). The samples analysed included 314 maternal samples and 318 infant samples from the intervention group, along with 286 maternal and 282 infant samples from the control group.

The baseline characteristics of the participants between the two groups were almost similar. The median age was 24·3 years in the intervention group and 24·6 years in the control group. The median duration of schooling was 8 years in both groups. The mean BMI was 22·6 kg/m^2^ in the intervention group and 22·3 kg/m^2^ in the control group (Table 1). During the 6-month intervention period, mothers consumed a full packet of snacks for an average of 155·41 ± 35·1 d, with 83·8 % of mothers consuming it for > 75 % of the days. Similarly, for MMNs, an average of 146·54 ± 34·5 d was recorded, with 78·7 % of mothers consuming them for > 75 % of the days^(14)^.

**Table 1:** Baseline characteristics of mother–infant dyads whose blood samples were analysed for micronutrient assay

Variables	Intervention (*n* 314)		Control (*n* 286)	
	Mean or *n*	sd or %	Mean or *n*	sd or %
Maternal characteristics				
Age, median (IQR), y	24·3	3·6	24·6	3·7
Duration of schooling, median (IQR), y	8	0–10	8	0–10
BMI, kg/m^2^	22·6	3·5	22·3	3·3
BMI < 18·5 kg/m^2^, *n* (%)	24	7·6	26	9·1
BMI 18·5–25·0 kg/m^2^, *n* (%)	218	69·4	198	69·2
BMI > 25·0 kg/m^2^, *n* (%)	72	22·9	62	21·7
Socio-demographic characteristics				
Wealth quintile, *n* (%)				
Poorest	62	19·8	59	20·6
Very poor	66	21·0	51	17·8
Poor	72	22·9	46	16·1
Less poor	50	15·9	65	22·7
Least poor	64	20·4	65	22·7
Infant characteristics at enrolment (age 0–7 d)	**Intervention** **(** ***n*** **318)**		**Control** **(** ***n*** **282)**	
Age at enrolment (days)	5·7	1·4	5·7	1·4
Birth weight, reported or documented, kg	4·1	2·8	3·7	2·5
Gestational age reported, weeks	38·7	1·8	38·5	1·9
Number of male infants, n (%)	156	49·1	146	51·8
BMI-Z* using WHO Growth Standards	–1·2	1·0	–1·2	0·9
LAZ*	–1·2	1·1	–1·3	1·1

IQR, interquartile range.

All values are mean (sd) unless specified otherwise.

* Using WHO 2006 Growth Standards.

There were significant differences in the micronutrient status of lactating mothers in the intervention and control groups. The mean serum ferritin concentration at 6 months was 47·4 ± 34·8 ng/ml in the intervention group and 32·4 ± 33·3 ng/ml in the control group, with an adjusted mean difference of 14·7 (95 % CI: 9·3, 20·2; *P* < 0·001). The intervention led to a significant 60 % (15 % *v*. 38 %; *P* < 0·001) reduction in the proportion of Fe deficiency anaemia. The intervention group also showed higher mean concentrations of serum retinol (3·4 ± 1·6 *v*. 2·9 ± 1·5 μmol/l; *P* < 0·001), serum folate (8·0 ± 4·9 *v*. 4·8 ± 2·9 ng/ml; *P* < 0·001) and serum vitamin D (10·6 ± 4·1 *v*. 9·6 ± 4·3 ng/ml; *P* < 0·01) compared with the control group. The prevalence of folate deficiency was also lower in the intervention group (28 % *v*. 47 %; *P* < 0·001). None of the mothers from the intervention group showed any statistically significant improvement in vitamin B_12_ or Zn micronutrient concentrations. The adjusted effect sizes for each micronutrient are summarised in Table 2. Compared with routine care, the risk of being deficient in Fe, vitamin D and folate reduced by 60 % (*P* < 0·001), 30 % (*P* < 0·01) and 50 % (*P* < 0·001), respectively, in the intervention group.

**Table 2:** Effect of maternal nutritional supplementation to lactating mothers on their micronutrient status at six months postpartum

Micronutrient status	N	Intervention		N	Control		Adjusted* mean difference/risk ratio (95 % CI)	
		Mean	sd		Mean	sd	Mean	95 % CI
Ferritin ng/ml	310	47·4	34·8	284	32·4	33·3	14·7***	9·3, 20·2
							0·4***	0·3, 0·5
Proportion deficit (%)		15·5			38·4			
Vitamin A μmol/l,	313	3·4	1·6	286	2·9	1·5	0·6***	0·3, 0·8
							0·1	0·02, 1·21
Proportion deficit (%)		0·3			2·1			
Vitamin B_12_ pg/ml,	309	352·4	129·4	285	345·8	128·2	6·9	–13·9, 27·7
							0·6	0·3, 1·1
Proportion deficit (%)		5·8			9·1			
Vitamin D ng/ml,	306	10·6	4·1	279	9·6	4·3	1·03**	0·3, 1·7
							0·7**	0·6, 0·8
Proportion deficit (%)		47·7			65·5			
Folate ng/ml,	296	8·0	4·9	284	4·8	2·9	3·3***	2·6, 3·9
							0·5***	0·4, 0·7
Proportion deficit (%)		28·7			46·9			
Zn µg/dl,	314	68·8	22·4	286	67·8	18·8	0·01	–0·02, 0·04
Proportion deficit (%)		53·1			53·7		0·9	0·8, 1·1

* Adjusted for baseline BMI and wealth quintile.

** *P* < 0.01.

*** *P* < 0.001.

All values are mean (sd), unless otherwise specified.

Maternal micronutrient proportion deficient calculated with cut-off defined as ferritin < 15 ng/ml, vitamin A < 0.7 μmol/l, vitamin B_12_ < 203 pg/ml, vitamin D < 12 ng/ml, folate < 4 ng/ml and Zn < 66 µg/dl.

**Fig. 2:**
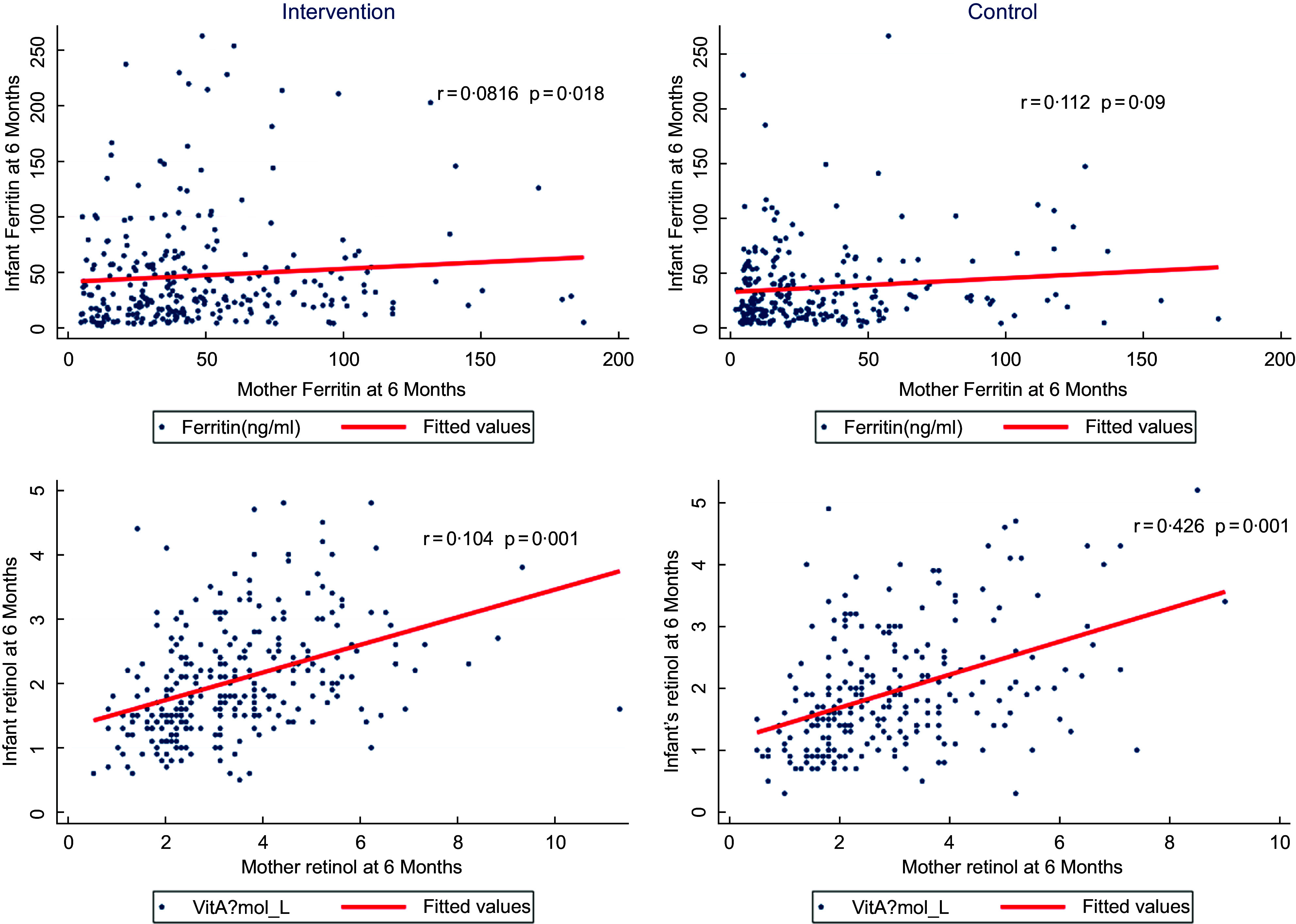
Correlation between maternal and infant levels of serum ferritin and retinol

Maternal nutritional supplementation was seen to be associated with increased concentrations of ferritin (mean difference of 8·9; 95 % CI: 1·2, 16·5; *P* < 0·01) and vitamin A (mean difference of 0·2; 95 % CI: 0·04, 0·4’ *P* < 0·01) in infants at 6 months postpartum; however, there were no statistically significant effects on other micronutrient concentrations. The adjusted effect sizes are summarised in Table 3. A further correlation analysis between maternal and infant serum ferritin, as well as between maternal and infant serum retinol (Fig. 2), suggests that there was a moderate correlation between changes in maternal retinol levels and infant retinol levels (*P* value < 0·001); however, the correlation was NS for serum ferritin (*P* > 0·05).

**Table 3:** Effect of maternal nutritional supplementation to mothers on infant micronutrient status at six months postpartum

Micronutrient status	*N*	Intervention		*N*	Control		Adjusted* mean difference/risk ratio (95 % CI)	
		Mean	sd		Mean	sd	Mean	95 % CI
Ferritin ng/ml, Mean (sd)	309	45·9	48·5	279	34·9	33·1	8·9**	1·2, 16·5
							0·8	0·5, 1·03
Proportion deficit (%)		19·2			23·3			
Vitamin A μmol/l, Mean (sd)	308	2·1	0·8	276	1·9	0·9	0·2**	0·04, 0·4
							0·4	0·1, 1·3
Proportion deficit (%)		2·6			4·7			
Vitamin B_12_ pg/ml, Mean (sd)	306	322·5	179·1	276	318·1	184·6	4·4	–30·1, 38·9
							0·9	0·7, 1·2
Proportion deficit (%)		27·8			31·2			
Vitamin D ng/ml, Mean (sd)	304	17·9	0·6	276	18·2	8·4	–0·7	–2·4, 0·9
							1·2	0·9, 1·6
Proportion deficit (%)		33·6			26·1			
Folate ng/ml, Mean (sd)	309	4·1	6·6	278	5·9	7·1	–1·5	–2·8, –0·3
							0·6	0·2, 1·4
Proportion deficit (%)		5·9			6·7			
Zn µg/dl, Mean (sd)	280	67·3	18·9	282	67·6	19·3	0·1	–4·2, 4·4
							1·1	0·8, 1·5
Proportion deficit (%)		29·1			28·2			

* Adjusted for gestational age, bmi *z* score at baseline, LAZ, wealth quintile.

** *P* < 0.01.

All values are mean (sd), unless specified otherwise.

Infant micronutrient proportion deficient was defined with cut-off as ferritin < 12 ng/ml, vitamin A < 0.7 μmol/l, vitamin B_12_ < 203 pg/ml, vitamin D < 12 ng/ml, folate < 4 ng/ml and Zn < 65 µg/dl.

## Discussion

Our study findings indicate that intervention positively improved the concentrations of ferritin, vitamin A, serum folate and vitamin D in mothers at 6 months postpartum, as well as on the concentrations of ferritin and vitamin A in infants. However, it did not significantly reduce the prevalence of other infant micronutrient deficiencies.

The importance of nutritional interventions for lactating mothers in resource-limited settings is widely acknowledged, but there is surprisingly little research on the topic. A Cochrane review conducted in 2019 reported MMN supplementation to pregnant women led to a 5 % reduction in preterm births (18 trials, 91 425 participants), an 8 % reduction in small-for-gestational age babies (17 trials, 57 348 participants) and a 12 % reduction in low birthweight newborns (18 trials, 68 801 participants)^(13)^ Another ramdomised controlled trial reported MMN supplementation to lactating mothers for nearly 3 months increased concentrations of maternal vitamins A, D and B_12_^(26)^. However, with a small sample size and inclusion of only non-anaemic women, caution must be applied in interpreting these findings. Our study demonstrates that 6-month nutritional supplementation for postpartum mothers significantly increases concentrations of most micronutrients, highlighting the potential of such interventions, especially in resource-limited settings.

Despite prior studies reporting the critical role of micronutrients such as Fe from conception to the postpartum period^(27,28)^, there is still a high prevalence of Fe deficiency anaemia among postpartum women in LMIC. This is concerning, considering the wider attention that postpartum anaemia has garnered even in developed countries due to its adverse effects on maternal health, including depression, obesity and general fatigue^(29)^. Encouragingly, our study found a significant reduction in maternal Fe deficiency anaemia among lactating mothers who received the nutritional supplementation, aligning with the results of a previous study among adolescent lactating mothers (*N* 36)^(30)^.

Vitamin A or retinol is required during the postpartum stage for the production of colostrum, transition milk and mature milk^(31)^. While a recent study found that single high-dose supplementation did not increase the concentration of vitamin A in mature breast milk, our study found a significant reduction in vitamin A deficiency among lactating mothers in the supplementation group^(32)^.

Our results also highlight the positive impact of maternal nutritional supplementation on folate and vitamin D deficient lactating mothers. In developed countries, lactating mothers typically have adequate folate levels due to their consumption of a diet rich in folate, owing to their high consumption of folate-rich diet^(33–35)^. However, in developing countries where the dietary intake is inadequate, supplementation should be considered. Although breast milk is not a rich source of vitamin D and lactation is not believed to deplete the vitamin D reserves in mothers, our study found that supplementing micronutrients reduced the prevalence of vitamin D deficiency at 6 months postpartum^(36)^. Existing evidence indicates that vitamin D supplementation has a dose-dependent response on maternal and infant vitamin D concentrations^(37)^.

While single micronutrient supplementation among pregnant women has improved child and maternal health outcomes, its impact during lactation is less explored^(38)^. A trial in India showed that vitamin B_12_ supplementation during pregnancy and early lactation enhanced the postpartum vitamin B_12_ status of mothers^(39)^. Despite substantial evidence supporting MMN supplementation during pregnancy to reduce fetal growth restriction and low birth weight, research on its benefits during lactation is limited^(40,41)^. Our study shows clear benefits of such interventions in enhancing the micronutrient status during the critical lactation period.

Our study revealed that maternal supplementation marginally increased the serum retinol levels in infants. However, it did not influence the levels of folate, vitamin D and Zn in infants. Retinol is a fat-soluble vitamin that can be secreted into breast milk and transferred to the infant^(42)^. However maternal supplementation did not decrease infant B_12_ deficiency. Community-based randomised controlled trials conducted in similar settings have indeed reported improvements in infant B_12_ concentrations when maternal micronutrient supplementation was initiated in early pregnancy and continued through lactation. This suggests that a comprehensive approach, starting in early pregnancy and continuing through lactation, may effectively enhance infant B_12_ levels^(43,44)^.

The results of this study have important implications for establishing dietary guidelines for breast-feeding mothers in LMIC. More evidence from randomised trials with direct infant supplementation is needed for policy recommendations. The lack of significant improvement in certain micronutrients in infants at 6 months could potentially be attributed to various factors, including the complex interplay between maternal nutrition, breast milk composition, infant feeding practices and the specific bioavailability and utilisation of these micronutrients by the growing infant, highlighting the need to better understand these dynamics and optimise interventions for improved infant micronutrient status.

Our study did not assess C-reactive protein, which could have provided insights into the potential influence of inflammation on micronutrient status. Furthermore, the calculation of difference-in-differences was not possible due to the absence of a pre-intervention data point, despite the availability of dietary data at 3 months of infant age, as published in the main paper. However, we have dietary intake data of mothers assessed using 24-h dietary recall at 3 mo of infant age in both the intervention and control group (see online supplementary material, Supplementary Table 1). In general, the intervention group has slightly higher intake of most nutrients compared with the control group. Despite these limitations, our study’s strength lies in its randomised design and relatively larger sample size, providing evidence on the impact of MMN supplementation on maternal and infant outcomes. With the current sample size, we achieved a statistical power of 85 % and a 95 % confidence level, to detect a minimum difference of 0·25 sd in the micronutrient status between the groups.

This study suggests that MMN supplementation during lactation may be beneficial in reducing maternal micronutrient deficiencies, compared with routine Fe and folic acid supplementation alone. This is particularly important in LMIC where diets often lack nutrient-rich and animal-source proteins^(12,45)^. While our findings suggest potential benefits of maternal nutritional supplementation on the micronutrient status of lactating mothers and their infants, we recognise that implementing such interventions on a larger scale would involve significant costs. These could include the direct costs of the supplements, as well as indirect costs related to distribution, monitoring and adherence. A study conducted in similar LMIC settings found that replacing Fe-folic acid supplementation with MMN supplements for pregnant women could be cost-effective in terms of cost per death averted and cost per disability-adjusted life year averted, potentially saving lives and reducing lifelong disabilities^(46)^.

By continuing to provide essential nutrients during the transition from pregnancy to lactation, healthcare providers can help support the overall health and well-being of both the mother and the baby^(13,47–49)^. While the metabolic interaction of different micronutrients when supplemented concurrently is still unknown, our findings suggest potential benefits to mothers. We also observed a modest beneficial effect on ferritin and vitamin A concentrations in infants at 6 months, which could positively impact population health.

In conclusion, the findings of this study provide important insights into the impact of additional nutrition through healthy snacks and MMN supplementation on the micronutrient status of lactating mothers and their infants. The results demonstrate significant improvements at 6 months in the concentrations of ferritin, vitamin A, serum folate and vitamin D in mothers postpartum, as well as in the concentrations of ferritin and vitamin A in infants. These positive effects highlight the potential of maternal nutritional interventions to enhance micronutrient status during the critical lactation period. Further exploration is warranted to investigate specific strategies that can effectively address micronutrient deficiencies and optimise interventions to improve infant micronutrient status.

## Supporting information

Manapurath et al. supplementary material 1 Manapurath et al. supplementary material

Manapurath et al. supplementary material 2 Manapurath et al. supplementary material

## Data Availability

Society for Applied Studies, India is a collaborator in the Healthy Birth, Growth, and Development Knowledge Integration (HBGDKi) initiative launched by the Bill & Melinda Gates Foundation and data generated will be shared as part of HBGDKi repository (https://github.com/HBGDki). The data described in the manuscript will not be made freely available in the public domain but will be shared as part of the HBGDKi repository. Any individual requests, if accompanied by a detailed proposal describing the research question and a brief note on methods may be considered on a case-by-case basis. Such request should be sent to Dr. Sunita Taneja (sunita.taneja@sas.org.in).

## Supplementary material

For supplementary material accompanying this paper visit https://doi.org/10.1017/S1368980024001824
