# Supplementary material for: Impact of nutritional and multiple micronutrients supplementation to lactating mothers 6 months postpartum on the maternal and infant micronutrient status: a randomised controlled trial in Delhi, India
Source: Public Health Nutr. 2024 Sep 26;27(1):e179. doi: 10.1017/S1368980024001824 (PMC11504856; doi:10.1017/S1368980024001824)
Supplement: Manapurath et al. supplementary material 2 — Manapurath et al. supplementary material [file S1368980024001824sup002.doc]

***Table 1: Comparative Analysis of Dietary Intake among Intervention and Control Group Mothers at 3 Months of Infant Age***

| **Variable** | **Intervention** | **Control** |
| --- | --- | --- |
| Energy, kcal | 2247 (775) | 2088 (874) |
| Carbohydrates, g | 304 (115) | 284 (130) |
| Proteins, g | 70 (26) | 64 (27) |
| Fats, g | 72 (37) | 69 (41) |
| Folate, µg | 222.1(112) | 211.1(119.3) |
| Retinol, µg | 106.3(116.2) | 106.4(111.2) |
| Vitamin D, µg | 0.7 (0.5) | 0.7 (1.2) |
| Zinc, mg | 8.1 (3.4) | 8.5 (3.3) |
| Iron, mg | 13.1 (5.5) | 12.4 (5.6) |
